# Effects of Exercise on Spinal Deformities and Quality of Life in Patients with Adolescent Idiopathic Scoliosis

**DOI:** 10.1155/2015/123848

**Published:** 2015-10-25

**Authors:** Shahnawaz Anwer, Ahmad Alghadir, Md. Abu Shaphe, Dilshad Anwar

**Affiliations:** ^1^Rehabilitation Research Chair, Department of Rehabilitation Sciences, College of Applied Medical Sciences, King Saud University, Riyadh 11433, Saudi Arabia; ^2^Dr. D. Y. Patil College of Physiotherapy, Dr. D. Y. Patil Vidyapeeth, Pune, India; ^3^Department of Physiotherapy, College of Applied Medical Sciences, Jazan University, Saudi Arabia; ^4^Department of Orthopedics, JNMC, AMU, Aligarh, India

## Abstract

*Objectives*. This systematic review was conducted to examine the effects of exercise on spinal deformities and quality of life in patients with adolescent idiopathic scoliosis (AIS). *Data Sources*. Electronic databases, including PubMed, CINAHL, Embase, Scopus, Cochrane Register of Controlled Trials, PEDro, and Web of Science, were searched for research articles published from the earliest available dates up to May 31, 2015, using the key words “exercise,” “postural correction,” “posture,” “postural curve,” “Cobb's angle,” “quality of life,” and “spinal deformities,” combined with the Medical Subject Heading “scoliosis.” *Study Selection*. This systematic review was restricted to randomized and nonrandomized controlled trials on AIS published in English language. The quality of selected studies was assessed by the PEDro scale, the Cochrane Collaboration's tool, and the Grading of Recommendations Assessment, Development, and Evaluation System (GRADE). *Data Extraction*. Descriptive data were collected from each study. The outcome measures of interest were Cobb angle, trunk rotation, thoracic kyphosis, lumbar kyphosis, vertebral rotation, and quality of life. *Data Synthesis*. A total of 30 studies were assessed for eligibility. Six of the 9 selected studies reached high methodological quality on the PEDro scale. Meta-analysis revealed moderate-quality evidence that exercise interventions reduce the Cobb angle, angle of trunk rotation, thoracic kyphosis, and lumbar lordosis and low-quality evidence that exercise interventions reduce average lateral deviation. Meta-analysis revealed moderate-quality evidence that exercise interventions improve the quality of life. *Conclusions*. A supervised exercise program was superior to controls in reducing spinal deformities and improving the quality of life in patients with AIS.

## 1. Introduction

Adolescent idiopathic scoliosis (AIS) is a structural deformity of the spine with 3-dimensional deformation, including lateral shift and vertebral rotation affecting children at puberty [1, 2]. The predisposing factors are genetic predisposition; connective tissue abnormalities; and skeletal, muscular, and neurological disturbances during growth. However, the exact cause remains unknown [3]. In the general population, the prevalence of AIS is about 2.5% with a Cobb angle of >10 degrees [2, 3]. A variety of risk factors may result in higher curve progression, including female gender, age of 10–12 years, absence of menarche, presence of thoracic curves, curve size at presentation >25 degrees, Risser sign of 0-1, and residual growth potential [2–5].

The primary goal of rehabilitation for AIS is to reduce the progression of curves thereby decreasing the risk of secondary impairment, including back pain, breathing problems, and cosmetic deformities, and improve the quality of life [3, 6]. Exercise plays a vital role in reducing curve progression and improving quality of life in AIS. Patients with thoracic curves ≤25 degrees and thoracolumbar or lumbar curves ≤20 degrees can effectively manage with exercise alone, whereas patients with thoracic curves of 25–50 degrees and thoracolumbar or lumbar curves of 20–40 degrees require bracing along with exercise [3, 7–9].

In a previous review, Negrini et al. (2008) reported that exercise had beneficial effects on the rate of progression and Cobb angle. They also found positive effects of exercise in reducing brace prescriptions [10]. More recently, Negrini et al. [11] reviewed the best available evidence regarding the rehabilitation approach to AIS and reported that the Society on Scoliosis Orthopaedic and Rehabilitation Treatment (SOSORT) had the best evidence-based guidelines. Low evidence was found for conservative treatment because out of 65 recommendations none had strong evidence (level I), 2 had moderate evidence (level II), and the remainder had weak evidence. Recently, Romano et al. conducted a Cochrane systematic review to evaluate the efficacy of scoliosis-specific exercise (SSE) in adolescent patients with AIS [12] and reported a lack of high-quality evidence to recommend the use of SSE for AIS. They identified one low-quality study that found the use of exercise more effective than electrostimulation, traction, and postural training to avoid scoliosis progression and one very low-quality study that found the use of SSE more effective than traditional physiotherapy for reducing brace prescriptions. Similarly, in a systematic review, Negrini et al. [13] reported lack of solid evidence for or against the effectiveness of physical exercise for reducing curve progression in AIS. In addition, Mordecai and Dabke reported low-quality evidence for the effect of exercise in the treatment of AIS [8].

To date, no systematic review has examined the effects of exercise on quality of life in patients with AIS. Therefore, more evidence is required regarding the effects of exercise on curve reduction and improvement in quality of life in AIS is required. The aim of this systematic review was to evaluate the effects of an exercise program on spinal deformities and quality of life.

## 2. Methods

### 2.1. Data Sources

Electronic databases, including Pubmed, CINAHL, Embase, Scopus, Cochrane Register of Controlled Trials, PEDro, and Web of Science, were searched for published studies using the keywords “postural correction,” “postural curve,” “Cobb angle,” “quality of life,” and “spinal deformities” combined with the Medical Subject Heading “scoliosis” and “exercise.” The bibliographical search was restricted to randomized and nonrandomized controlled trials published in English language from the earliest available dates up to May 31, 2015 (see Search Strategy). Original authors were contacted and asked to provide the full text of potential papers that were not accessible. Two independent reviewers (Shahnawaz Anwer and Md. Abu Shaphe) selected the studies based on titles and abstracts, excluding those articles not related to the objectives of this review. Consensus between the reviewers was obtained through discussion.

*Search Strategy*

(1) Pubmed:
   #1 Scoliosis [MeSH Terms],
   #2 Exercise [MeSH Terms],
   #3 Postural correction [MeSH Terms],
   #4 Cobb angle [MeSH Terms],
   #5 Postural curve [MeSH Terms],
   #6 Quality of life [MeSH Terms],
   #7 [#1 AND (#2 OR #3)],
   #8 [#1 AND (#4 OR #5)],
   #9 [#1 AND (#2 OR #6)],
   Limits: Comparative study, randomized controlled trial;
(2) Cochrane Register of Controlled Trials:
   #1 Scoliosis [MeSH Terms],
   #2 Exercise [MeSH Terms],
   #3 Postural correction [MeSH Terms],
   #4 Cobb angle [MeSH Terms],
   #5 Postural curve [MeSH Terms],
   #6 Quality of life [MeSH Terms],
   #7 [#1 AND (#2 OR #3)],
   #8 [#1 AND (#4 OR #5)],
   #9 [#1 AND (#2 OR #6)],
   Limits: Trials;
(3) PEDro:
   
*∗* Advance Search,
   Title or abstract: Scoliosis, exercise, Cobb angle, Quality of life,
   Method: Clinical trial;
(4) Web of Science:
   
*∗* Advance Search,
   #1 Scoliosis [MeSH Terms],
   #2 Exercise [MeSH Terms],
   #3 Cobb angle [MeSH Terms],
   #4 Quality of life [MeSH Terms],
   #5 (#1 AND #2),
   #6 (#1 AND #3),
   #7 (#1 AND #2 AND #4),
   Limits: language (English), Document type (Article).

### 2.2. Study Selection

Studies were included on the basis of the following criteria: randomized and nonrandomized controlled methodology; subjects with AIS in the age group of 10–19 years; studies comparison of exercise with other interventions or controls; and outcome measures of radiological deformity (i.e., Cobb angle), surface deformities (including trunk rotation, thoracic kyphosis, lumbar lordosis, and average lateral deviation), and quality of life. Studies were excluded if subjects were >19 years of age, interventions did not include exercise or compare exercise with a control, or published results were in abstract form only. Final study selection was achieved through discussion and consensus between the two reviewers.

### 2.3. Data Extraction

The selected studies were screened by 2 independent reviewers (Shahnawaz Anwer and Md. Abu Shaphe). One of the reviewers had prior experience using the extraction form, systematic review methodology, and quality appraisal tools, including the PEDro and Cochrane databases, and had published two systematic reviews and a meta-analysis. The other reviewer was trained beforehand in the use of the extraction form, systematic review methodology, and quality appraisal tools, including the PEDro and Cochrane databases. The following items were extracted: author/year, design of the study, subject's characteristics, age, sex, sample size, details of exercise program (type, duration, dose, and frequency), outcomes, and conclusions. The two reviewers discussed the data with each other or consulted with a third reviewer (Ahmad Alghadir) to reach consensus. Agreement between the two reviewers was obtained using unweighted kappa (*κ*) statistics. Mean and standard deviation of the baseline and final end point scores for the Cobb angle, trunk rotation, thoracic kyphosis, lumbar lordosis, lateral deviation, and function were extracted from included studies. The mean change score (final score minus baseline score) for each outcome measure was calculated for each intervention. The standardized mean difference (SMD) for all the outcomes was computed [14].

Cohen's categories were used to define the magnitude of the effect size with values of <0.5 as a small effect size; ≥0.5 and ≤0.8, as a medium effect size; and >0.8, as a large effect size [15]. A fixed-effects meta-analysis was conducted to determine the overall effect size of exercise. The *z* statistic was used to test the significance of an overall effect. Cochran's *Q* statistic and Higgins' *I* ^2^ statistic were used to determine statistical heterogeneity between studies [14]. All statistics were computed using Comprehensive Meta-Analysis software [16].

### 2.4. Assessment of Methodological Quality

The 11-item PEDro scale was used to assess the quality of included studies by two independent reviewers (Shahnawaz Anwer and Md. Abu Shaphe) [17]. A study with a score ≥6 was considered high-quality as reported previously [18]. In addition, the Cochrane Collaboration's tool was used to assess the risk of bias. Sequence generation, allocation concealment, blinding, completeness of outcome data, and absence of selective outcome reporting were also assessed. Risk of bias was classified as low, unclear, or high in each domain [19]. Agreement between the two reviewers in regard to the PEDro and Cochrane tools was made using unweighted kappa (*κ*) statistics.

The quality of evidence was determined using the Grading of Recommendations Assessment, Development, and Evaluation System (GRADE) for each meta-analysis [20]. This method involves downgrading evidence from high-quality to moderate-quality to low-quality and to very low-quality using some factors. If the majority of studies (more than 50%) in the meta-analysis had a PEDro score <6 or had more than low levels of statistical heterogeneity between the studies (*I* ^2^ > 25%) [21] or if the studies had large confidence intervals suggestive of a small number of subjects in the studies, then the evidence would be downgraded, for example, from high- to moderate-quality. In the presence of serious methodological flaws, for example, if all studies in the meta-analysis had low PEDro scores (<6) with no allocation concealment and blinding, the evidence would be double downgraded (e.g., from high- to low-quality). The criteria for the grade applied to each meta-analysis are explained as a footnote.

## 3. Results

### 3.1. Identified Studies

**Figure 1:**
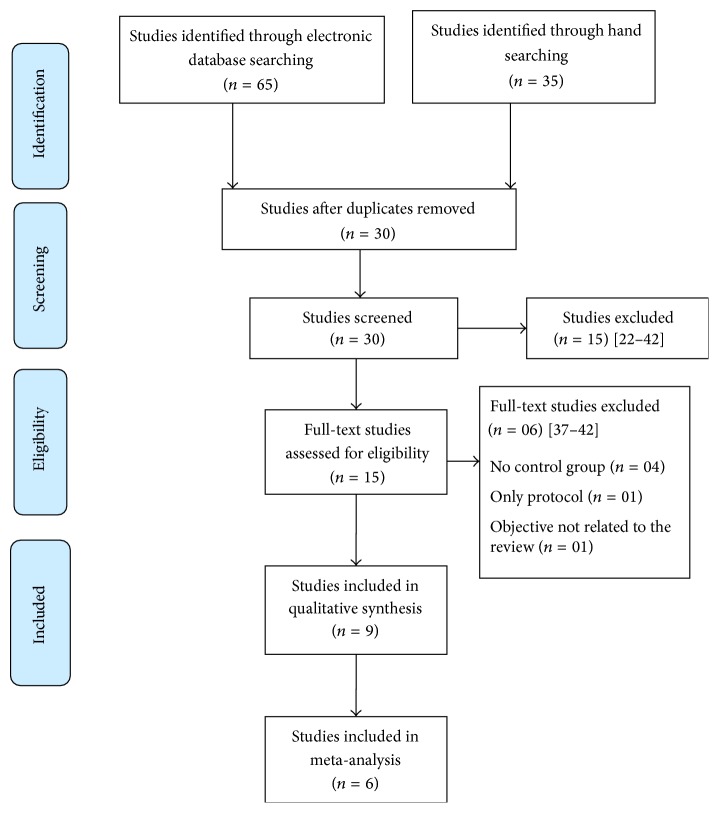
Flow diagram of the study procedure.

The abstracts of 30 studies were assessed for eligibility. Twenty-one studies [22–42] were eliminated because they did not match the inclusion criteria or were not available in full text (Figure 1). A total of 9 studies were included in the quality assessment phase [43–51].

### 3.2. Quality Assessment of Study

The 9 included studies had an average PEDro score of 5.7/10, as illustrated in Table 1. Agreement between reviewers was good (unweighted *κ* = 0.79). However, multiple sources of bias in these studies may have skewed the results. The most common shortcomings were lack of randomization [46–49, 51], lack of concealed allocation [46–49, 51], and lack of blinding (patient, therapist, or assessor) [43–51]. The most adhered ones to items on the PEDro scale were baseline comparability, follow-up, intention-to-treat analysis, measurements of variability, and between-group comparisons, which were evident in almost all the trials.

Agreement between the reviewers was excellent (unweighted *κ* = 0.87) in assessing risk of bias across studies. Details of the risk of bias assessment of included studies are given in Table 2. The overall risk of bias assessment indicated that the risk of bias was low in 1 study [43], high in 5 studies [46–49, 51], and unclear in 3 studies [44, 45, 50]. The most common shortcomings were lack of blinding [47–49, 51], lack of concealment [46–49, 51], and inadequate random sequence generation [46–49, 51].

### 3.3. Characteristics of Study Populations

Table 3 details participant characteristics. The sample size for whole study groups ranged from 30 to 252, with the mean age varying from 12 to 15 years. In most of the studies, the majority of participants with AIS were female [43, 44, 46–49, 51]. Most of the studies used the Cobb angle and Risser sign as inclusion criteria for participants with AIS [43–49, 51].

### 3.4. Training Protocol

Table 3 summarizes the training protocol. Three studies compared the Scientific Exercise Approach to Scoliosis (SEAS.02) exercises with controls [47, 48, 51], 1 study compared active self-correction and task-oriented exercises with controls [43], 1 study compared Schroth exercises with controls [44], 1 study compared forward head correction and traditional exercise with controls [45], 1 study compared the 3D corrective spinal technique with controls [46], 1 study compared physiologic exercise program and scoliosis intensive rehabilitation (SIR) with control [49], and 1 study compared passive transverse force and SIR with a control group [50]. In all included studies, the control group received usual care or performed a traditional exercise program. Only one study [43] had a report of an adverse effect which was a minor temporary worsening of pain.

### 3.5. Outcome Measures

Six studies used the Cobb angle [43, 44, 46–48, 51], 5 studies used the angle of trunk rotation [43, 44, 47, 48, 51], 3 studies used the thoracic kyphosis angle [45, 46, 49], 2 studies used the lumbar lordosis angle [45, 46], and 3 studies used the average lateral deviation [45, 49, 50] to measure various spinal deformities. Radiographic methods were used to measure the Cobb angle in all six included studies, and a Scoliometer was used to measure the angle of trunk rotation in the 5 included studies. Two studies used a Formetric device to measure thoracic kyphosis [45, 49], and 1 study used a radiographic method for this measurement [46]. One study used a Formetric device to measure lumbar lordosis [45], whereas the other study used a radiographic method for this measurement [46]. Average lateral deviation was measured with a Formetric device in all 3 included studies. Two studies used the Scoliosis Research Society-22 patient questionnaire (SRS-22) [43, 46], 1 study used SRS-23 [44], and another used the Functional Rating Index to measure health related quality of life [45]. The Functional Rating Index is a patient-rated scale composed of 10 items including 4 subscales: pain, sleep, work, and daily activity [52]. The subscales include 3 domains of the World Health Organization International Classification of Functioning, Disability, and Health (WHO-ICF) such as activity limitations with 6 items (personal care, travel, recreation, lifting, walking, and standing), impairment with 3 items (pain frequency, pain intensity, and sleep), and participation restriction with 1 item (work). Each item was scored on a 5-point scale ranging from 0 (no pain or maximum ability) to 4 (maximum pain or disability). The possible score ranges from 0% (no disability) to 100% (severe disability).

### 3.6. Effect of Exercise on Spinal Deformities

**Figure 2:**
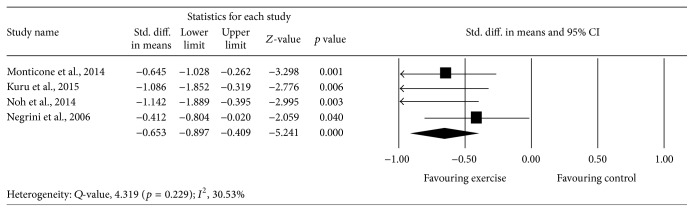
Effect of exercise on the Cobb angle.

**Figure 3:**
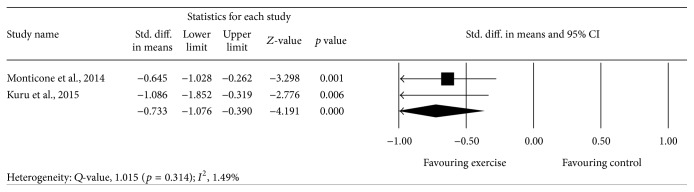
Effect of exercise on the angle of trunk rotation.

**Figure 4:**
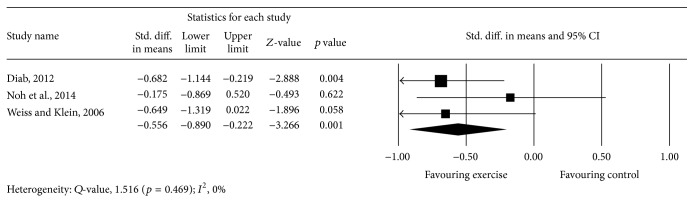
Effect of exercise on the thoracic kyphosis angle.

**Figure 5:**
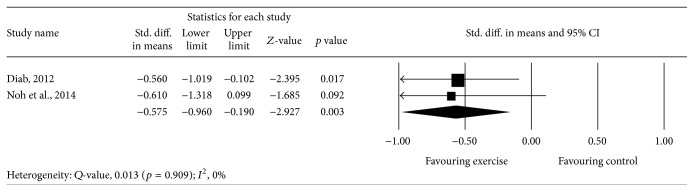
Effect of exercise on the lumbar lordosis angle.

**Figure 6:**
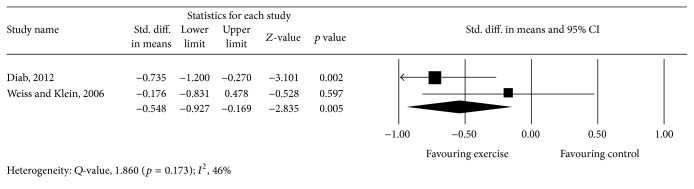
Effect of exercise on the average lateral deviation.

**Figure 7:**
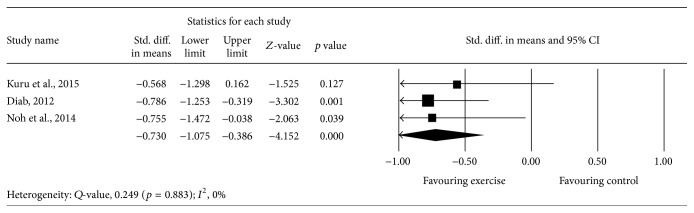
Effect of exercise on the quality of life.

Table 4 gives details of the results of the exercise and control group in included studies. Data syntheses of included studies are given in Table 5 and Figures 2–7. Meta-analysis of 4 studies [43, 44, 46, 47] provided moderate-quality evidence with a significant effect size point estimate across the 4 included studies (*p* = 0.000), with an overall medium effect size point estimate of 0.65 (95% CI, −0.89 to −0.40) based on a fixed-effects model that favored exercise compared with controls in reducing the Cobb angle (Figure 2).

Meta-analysis of 2 studies [43, 44] provided moderate-quality evidence with a significant effect (*p* = 0.000), with an overall medium effect size point estimate of 0.73 (95% CI, −1.07 to −0.39) based on a fixed-effects model that favored exercise compared with controls in reducing the angle of trunk rotation (Figure 3).

Meta-analysis of 3 studies [45, 46, 49] provided moderate-quality evidence with a significant effect size point estimate across the 3 included studies (*p* = 0.001), with an overall medium effect size point estimate of 0.55 (95% CI, −0.89 to −0.22) based on a fixed-effects model that favored exercise compared with controls in reducing the thoracic kyphosis angle (Figure 4).

Meta-analysis of 2 studies [45, 46] provided moderate-quality evidence with a significant overall effect (*p* = 0.003), with an overall medium effect size point estimate of 0.57 (95% CI, −0.96 to −0.19) based on a fixed-effects model that favored exercise compared with controls in reducing lumbar lordosis (Figure 5).

Meta-analysis of 2 studies [45, 50] provided low-quality evidence with a significant overall effect (*p* = 0.005), with an overall medium effect size point estimate of 0.54 (95% CI, −0.92 to −0.16) based on a fixed-effects model that favored exercise compared with controls in reducing average lateral deviation (Figure 6).

### 3.7. Effect of Exercise on Quality of Life

Meta-analysis of 3 studies [44–46] provided moderate-quality evidence with a significant effect size point estimate across the 3 included studies (*p* = 0.000), with an overall medium effect size point estimate of 0.73 (95% CI, −1.07 to −0.03) based on a fixed-effects model that favored exercise compared with controls in improving the quality of life (Table 5 and Figure 7).

## 4. Discussion

This systematic review investigated current available evidence on the effects of an exercise program on spinal deformities and quality of life in patients with AIS. The review evaluated 9 studies, including a total of 768 participants.

Among the 9 studies evaluated using the PEDro scale [17], 6 were considered of high methodological quality [43–48]. The overall risk of bias assessment showed that 5 studies had a high risk of bias [46–49, 51], and 1 study had a low risk of bias [43], while others had an unclear risk of bias [44, 45, 50]. More than half of the studies failed to perform blinding and (Table 2).

The results of the present systematic review provide moderate-quality evidence for exercise intervention with a medium effect size for reducing the Cobb angle, angle of trunk rotation, thoracic kyphosis angle, and lumbar lordosis angle and improving the quality of life in patients with AIS. Similarly, a systematic review conducted by Fusco et al. [53] reported improvement in the Cobb angle following a regime of exercise. In another review, Negrini et al. [10] confirmed the efficacy of exercises in reducing the progression of deformity and Cobb angles in patients with AIS. In contrast, Mordecai and Dabke [8] reported poor quality evidence supporting the use of an exercise program in the management of AIS, and a Cochrane review conducted by Romano et al. [12] revealed a lack of high-quality evidence to recommend the use of scoliosis-specific exercises to reduce the progression of AIS.

All previous reviews were focused on the effects of exercise on the Cobb angle and brace prescriptions in patients with AIS [8, 10, 53]. However, in the present review, in addition to the Cobb angle, other surface spinal deformities such as trunk rotation, thoracic kyphosis, lumbar lordosis, average lateral deviation, and quality of life were measured. Moreover, in previous reviews, only Romano et al. [12] performed a meta-analysis of the effects of scoliosis-specific exercises to reduce the progression of AIS.

In the present review, 3 studies compared SEAS.02 exercise with a control group and reported that SEAS.02 exercises were superior to control conditions for reducing spinal deformities and the progression of scoliosis [47, 48, 51]. Another 6 studies included in the present review compared 6 different exercise protocols with traditional spinal exercises. All these studies reported significant reduction of spinal deformities and improvement in quality of life as compared with traditional spinal exercise [43–46, 49, 50].

This review had several limitations. Inclusion criteria were not well defined in the included studies, and the majority of the included studies were nonrandomized. Additionally, lack of blinding, lack of concealed allocation, and variations in exercise protocols are significant limitations in the current published literature. Moreover, different types of exercise have different intensities and may induce different effects, and the presence of heterogeneity in exercise protocols prevents conclusive results. For example, the total intervention duration varied between 2 weeks [43] and 4 months [46] and sample size in the included studies varied from 30 [44] to 252 [50]. Another limitation of the present review was the inclusion of only studies published in English, which might have created some selection bias. In addition, most of the included studies did not clarify what types of exercises are found in the usual care.

## 5. Conclusions

Moderate-quality evidence suggests that an exercise program is superior to controls in reducing the Cobb angle, angle of trunk rotation, thoracic kyphosis angle, and lumbar lordosis angle and improving the quality of life in patients with AIS; and the low-quality evidence suggests that an exercise program is superior to controls in reducing average lateral deviation in patients with AIS. However, the presence of heterogeneity in exercise protocols and poor methodological quality limit the validity of these results.

## Figures and Tables

**Table 1:** Methodological classification assessed by PEDro scale.

Criteria	Monticone et al. (2014) [43]	Kuru et al. (2015) [44]	Diab (2012) [45]	Noh et al. (2014) [46]	Negrini et al. (2006) [47]	Negrini et al. (2006) [48]	Weiss and Klein (2006) [49]	Weiss et al. (2002) [50]	Negrini et al. (2008) [51]	Cumulative score^*∗*^
Random allocation?	Yes	Yes	Yes	No	No	No	No	Yes	No	4
Concealed allocation?	Yes	Yes	Yes	No	No	No	No	Yes	No	4
Baseline comparability?	Yes	Yes	Yes	Yes	Yes	Yes	Yes	No	Yes	8
Blind participants?	Yes	No	No	No	No	No	No	No	No	1
Blind therapists?	No	No	No	No	No	No	No	No	No	0
Blind assessors?	Yes	No	No	Yes	No	No	No	No	No	2
Follow-up?	Yes	Yes	Yes	Yes	Yes	Yes	Yes	Yes	Yes	9
Intention-to-treat analysis?	Yes	Yes	Yes	Yes	Yes	Yes	Yes	Yes	Yes	9
Group comparisons?	Yes	Yes	Yes	Yes	Yes	Yes	No	No	Yes	7
Point and variability measures?	Yes	Yes	Yes	Yes	Yes	Yes	Yes	No	No	7
Cumulative score	9	7	7	6	5	5	4	4	4	**5.7** ^†^

^*∗*^Out of the 10 total studies.

^†^Maximum score of 10.

**Table 2:** Risk of bias of included studies (yes, low risk of bias; no, high risk of bias).

Citations	Adequate sequence generation?	Allocation concealment?	Blinding?	Incomplete outcome data addressed?	Free of selective reporting?	Conclusions
Monticone et al. (2014) [43]	Yes	Yes	Yes	Yes	Yes	Low risk of bias
Kuru et al. (2015) [44]	Yes	Yes	Unclear	Yes	Yes	Unclear risk of bias
Diab (2012) [45]	Yes	Yes	Unclear	Yes	Yes	Unclear risk of bias
Noh et al. (2014) [46]	No	No	Yes	Yes	Yes	High risk of bias
Negrini et al. (2006) [47]	No	No	No	Yes	Yes	High risk of bias
Negrini et al. (2006) [48]	No	No	No	Yes	Yes	High risk of bias
Weiss and Klein (2006) [49]	No	No	No	Yes	Yes	High risk of bias
Weiss et al. (2002) [50]	Yes	Yes	Unclear	Yes	Yes	Unclear risk of bias
Negrini et al. (2008) [51]	No	No	No	Yes	Yes	High risk of bias

**Table 3:** Overview of selected studies in adolescent idiopathic scoliosis.

Study	Subjects	Mean age, years (male/female, %)	Design	Group	Duration	Adverse effects	Conclusions
Monticone et al. (2014) [43]	AIS Cobb angle: 10–25 degrees Risser sign: <2	Group 1: 12.5 (29/71) Group 2: 12.4 (25/75)	RCT	1: active self-correction and task-oriented exercise (*n* = 55) 2: control: traditional spinal exercise (*n* = 55)	60-minute outpatient sessions once a week and 30-minute home exercise sessions twice a week for 2 weeks Follow-up: 12 months	Minor temporary pain worsening (*n* = 11 exp. group; *n* = 14 control group)	The active self-correction and task-oriented exercise was superior to traditional spinal exercises in reducing spinal deformities

Kuru et al. (2015) [44]	AIS Cobb angle: 10–60 degrees Risser sign: 0–3	Group 1: 12.9 (7/93) Group 2: 12.8 (13/87)	RCT	1: Schroth exercises (*n* = 15) 2: control (*n* = 15)	90-minute sessions thrice a week for 6 weeks Follow-up: 18 weeks	Not reported	Supervised Schroth exercise was superior to control group in reducing spinal deformities.

Diab (2012) [45]	AIS Cobb angle: 10–30 degrees Risser sign: 0–2	Group 1: 13.2 (53/47) Group 2: 14.5 (55/45)	RCT	1: forward head correction and traditional exercise (*n* = 38) 2: control: traditional exercise (*n* = 38)	3 sessions a week for 10 weeks Follow-up: 3 months	Not reported	A regime of forward head corrective exercise in addition to traditional exercises improved scoliotic posture and functional status.

Noh et al. (2014) [46]	AIS Risser sign: 0–4	Group 1: 13.8 (25/75) Group 2: 14.9 (13/87)	Retrospective Nonrandomized	1: 3D corrective spinal technique (*n* = 16) 2: control: traditional exercise (*n* = 16)	60-minute sessions 2-3 times per week for 3.5 to 4 months Follow-up: no	Not reported	A regime of corrective spinal technique was superior to traditional exercise in reducing most of the spinal deformities and improved quality of life.

Negrini et al. (2006) [47]	AIS Cobb angle: >15 degrees Risser sign: 0–3	Group 1: 13.3 (17/83) Group 2: 13.6 (13/87)	Prospective nonrandomized controlled study	1: SEAS.02 (*n* = 40) 2: control (*n* = 70)	1: 1.5-hour sessions every 2-3 months with prosecution in a facility near home for 40 minutes twice a week and 1 exercise daily for 5 minutes 2: performing exercises 2-3 times a week for 45 to 90 minutes Follow-up: no	Not reported	SEAS.02 exercises were superior to control group for reducing spinal deformities.

Negrini et al. (2006) [48]	AIS Cobb angle: >15 degrees Risser sign: 0–3	Group 1: 12.7 (22/78) Group 2: 12.1 (24/76)	Prospective nonrandomized controlled study	1: SEAS.02 (*n* = 23) 2: control (*n* = 25)	1: 1.5-hour sessions every 2-3 months with prosecution in a facility near home for 40 minutes twice a week and 1 exercise daily for 5 minutes 2: performing exercises 2-3 times a week for 45 to 90 minutes Follow-up: no	Not reported	SEAS.02 exercises were superior to control group for reducing spinal deformities.

Weiss and Klein (2006) [49]	AIS Cobb angle: >20 degrees	Group 1: 15.3 (0/100) Group 2: 14.7 (0/100)	Prospective controlled study	1: SIR and physiologic exercise (*n* = 18) 2: control: SIR (*n* = 18)	1: 5 days a week (2 hours in the morning and evening each) for 4 weeks and additionally 90 minutes of physiologic exercise for 5 days a week on second or third week 2: 5 days a week (2 hours in the morning and evening each) for 4 weeks	Not reported	Physiologic exercise program in addition to SIR was superior to SIR alone for correcting lateral deviation.

Weiss et al. (2002) [50]	AIS Age group: 12 to 18 years	Group 1: 14.8 (NR) Group 2: 15.2 (NR)	RCT	1: SIR and PTF treatment (*n* = 126) 2: control: SIR (*n* = 126)	1: having 5-6 hours of in-patient intensive program and additionally 4–6 PTF treatment of 20 minutes for 4–6 weeks 2: having only 5-6 hours of in-patient intensive program Follow-up: no	Not reported	In-patient rehabilitation with PTF was superior to in-patient rehabilitation alone to correct scoliotic posture.

Negrini et al. (2008) [51]	AIS Cobb angle: >15 degrees Risser sign: 0–3	Group 1: 12.7 (29/71) Group 2: 12.1 (31/69)	Prospective controlled cohort study	1: SEAS.02 (*n* = 35) 2: control: usual physiotherapy (*n* = 39)	1: 1.5-hour sessions every 2-3 months with prosecution in a facility near home for 40 minutes twice a week and 1 exercise daily for 5 minutes 2: performing exercises 2-3 times a week for 45 to 90 minutes Follow-up: no	Not reported	SEAS.02 exercises were superior to control group for reducing progression of scoliosis.

AIS, adolescent idiopathic scoliosis; RCT, randomized controlled trial; SEAS.02, Scientific Exercise Approach to Scoliosis; SIR, scoliosis intensive rehabilitation; PTF, passive transverse force; SRS-22, Scoliosis Research Society-22.

**(a):** 

Citation	Outcomes	Exercise group					Control group					*p* values^*∗*^
		Pretest	Posttest	Follow-up	^*t*^ *D*	^*f*^ *D*	Pretest	Posttest	Follow-up	^*t*^ *D*	^*f*^ *D*	
Monticone et al. (2014) [43]	Cobb angle (degree)	19.3 (3.9)	14.0 (2.4)	14.3 (2.3)	−5.3 (0.6)	−4.9 (0.4)	19.2 (2.5)	20.9 (2.2)	22.0 (1.6)	1.7 (0.3)	2.8 (0.4)	<0.001^†^ <0.001^‡^ 0.861^#^ <0.001^##^
	Angle of trunk rotation (degree)	7.1 (1.4)	3.6 (1.1)	3.3 (1.1)	−3.5 (0.2)	3.7 (0.2)	6.9 (1.3)	6.6 (1.2)	6.5 (1.1)	−0.2 (0.1)	−0.4 (0.1)	<0.001^†^ <0.001^‡^ 0.403^#^ <0.001^##^
	SRS-22											
	Function (0–5)	3.8 (0.5)	4.7 (0.2)	4.8 (0.2)	0.89 (0.07)	1.0 (0.07)	3.9 (0.5)	4.0 (0.4)	3.9 (0.4)	0.09 (0.03)	0.01 (0.04)	<0.001^†^ <0.001^‡^ 0.404^#^ <0.001^##^
	Pain (0–5)	3.8 (0.4)	4.6 (0.3)	4.7 (0.2)	0.82 (0.05)	0.89 (0.06)	3.9 (0.5)	4.3 (0.3)	4.2 (0.4)	0.45 (0.06)	0.33 (0.06)	<0.001^†^ <0.001^‡^ 0.383^#^ <0.001^##^
	Image (0–5)	3.6 (0.6)	4.4 (0.3)	4.6 (0.3)	0.82 (0.07)	1.0 (0.08)	3.4 (0.6)	3.7 (0.5)	3.6 (0.4)	0.30 (0.03)	0.21 (0.04)	<0.001^†^ <0.001^‡^ 0.094^#^ <0.001^##^
	Mental health (0–5)	3.8 (0.6)	4.5 (0.3)	4.7 (0.2)	0.75 (0.07)	0.95 (0.08)	3.9 (0.6)	4.01 (0.5)	3.8 (0.4)	0.11 (0.03)	−0.1 (0.04)	<0.001^†^ <0.001^‡^ 0.433^#^ <0.001^##^
	Satisfaction	NR	4.8 (0.3)	4.9 (0.3)	NR	NR	NR	4.0 (0.5)	4.2 (0.5)	NR	NR	<0.001^##^

Kuru et al. (2015) [44]	Cobb angle (degree)	33.4 (8.9)	NR	30.87 (8.9)	NR	−2.53	30.3 (6.6)	NR	33.3 (6.6)	NR	3.13	0.397^#^ 0.006^###^
	Angle of trunk rotation (degree)	11.9 (5.2)	NR	7.66 (5.24)	NR	−4.23 (4.78)	8.4 (2.9)	NR	10.5 (4.21)	NR	2.06 (2.09)	0.106^#^ 0.000^###^
	SRS-23	3.9 (0.6)	NR	4.23 (0.7)	NR	0.33 (0.34)	4.1 (0.4)	NR	4.07 (0.4)	NR	−0.03 (0.23)	0.452^#^ 0.131^###^

Diab (2012) [45]	Thoracic kyphosis	8.9 (0.9)	10.4 (1.1)	10 (1.05)	NR	NR	8.8 (1.5)	9 (1.8)	8.9 (1.7)	NR	NR	0.001^##^ 0.004^###^
	Lumbar lordosis	18.6 (5.4)	21.6 (1.8)	20.9 (1.9)	NR	NR	18.1 (5.5)	20 (1.7)	19.2 (1.6)	NR	NR	0.01^##^ 0.017^###^
	Lateral deviation	16.8 (2.3)	14.3 (2.3)	14.7 (2.4)	NR	NR	15.1 (1.8)	14.5 (1.6)	15.5 (1.7)	NR	NR	0.001^##^ 0.002^###^
	Functional index	13.9 (1.7)	10.7 (0.9)	10 (0.9)	NR	NR	16.1 (1.7)	11.9 (0.8)	13.8 (1.9)	NR	NR	0.8^##^ 0.001^###^

**(b):** 

Citation	Outcomes	Exercise group			Control group			*p* values^*∗*^
		Pretest	Posttest	^*t*^ *D*	Pretest	Posttest	^*t*^ *D*	
Noh et al. (2014) [46]	Cobb angle (degree)	21.6 (10.1)	13.5 (12)	8.1 (4.5)	19 (7)	14.7 (7.2)	4.3 (2.1)	<0.001^†^ <0.001^‡^ 0.003^##^
	Thoracic kyphosis	26.7 (12.6)	25.5 (9.3)	1.2 (9.9)	24.3 (8.1)	24.5 (7.5)	−0.2 (7)	0.611^†^ 0.904^‡^ 0.625^##^
	Lumbar lordosis	52.8 (17.8)	47.7 (6.7)	5 (14.2)	45.6 (12.8)	49 (7.4)	−3.3 (13.4)	0.176^†^ 0.332^‡^ 0.095^##^
	SRS22							
	Function (0–5)	4.1 (2)	4.7 (1)	NR	4.4 (0.8)	4.6 (1)	NR	0.027^†^ 0.083^‡^ 0.931^#^ 0.216^##^
	Pain (0–5)	4.5 (2.4)	4.9 (1)	NR	3.8 (1.6)	4.6 (2.4)	NR	0.026^†^ 0.066^‡^ 0.140^#^ 0.190^##^
	Image (0–5)	3.3 (1.2)	4.2 (1)	NR	2.9 (0.8)	3.4 (1)	NR	0.011^†^ 0.102^‡^ 0.343^#^ 0.026^##^
	Mental health (0–5)	4 (3)	4.6 (1.4)	NR	3 (1.4)	4 (1.2)	NR	0.026^†^ 0.066^‡^ 0.228^#^ 0.121^##^
	Satisfaction	NR	5 (1)	NR	NR	4 (1)	NR	0.039^##^
	Total	3.8 (1.8)	4.5 (0.4)	NR	3.5 (1.1)	4.1 (1.4)	NR	0.012^†^ 0.066^‡^ 0.306^#^ 0.041^##^

Negrini et al. (2006) [47]	Cobb angle (degree)	30.6 (10.8)	NR	−5.7 (5.6)	31.3 (11.3)	NR	−3.4 (11.3)	<0.05^¶^

Negrini et al. (2006) [48]	Cobb angle (degree)	15.3 (5.4)	NR	−3.2 (6.2)	14.9 (6)	NR	NR	<0.05^¶^

Weiss and Klein (2006) [49]	Lateral deviation (mm)	15.4 (5.1)	13.1 (5)	2.31 (5.6)	13.5 (6.8)	13.1 (6.2)	0.32 (2.5)	0.1^†^ 0.6^‡^
	Thoracic kyphosis angle (degree)	46.5 (8)	45.8 (7.7)	NR	48.8 (11)	46.8 (10.2)	NR	>0.05^†^ >0.05^‡^

Weiss et al. (2002) [50]	Lateral deviation (mm)	NR	NR	1.50	NR	NR	0.94	0.030^†^ 0.103^‡^

Negrini et al. (2008) [51]	Cobb angle (degree)	NR	NR	−0.67	NR	NR	1.38	<0.05^†^ <0.05^‡^
	Angle of trunk rotation (degree)	NR	NR	0.12	NR	NR	0.52	>0.05^†^ >0.05^‡^

^†^Pretest versus posttest in exercise group; ^‡^pretest versus posttest in control group; ^#^between-group comparison at baseline; ^##^between-group comparison at posttest; ^###^between-group comparison at follow-up; ^*t*^ *D*, difference between pretest and posttest; ^*f*^ *D*, difference between pretest and follow-up; ^¶^comparison of mean difference between two groups; ^*∗*^significant at *p* < 0.05; NR, not reported.

**Table 5:** Meta-analyses of effect of exercise program.

Outcomes	Number of studies	Ratio of studies (PEDro <6)	Number of subjects	SMD [95% CI]	*I* ^2^	Quality of evidence (GRADE)
Cobb angle	4	25%	282	0.65 [−0.89, −0.40]	30.53%	Moderate^†^
Angle of trunk rotation	2	0%	140	0.73 [−1.07, −0.39]	1.49%	Moderate^‡^
Thoracic kyphosis angle	3	33%	144	0.55 [−0.89, −.22]	0%	Moderate^‡^
Lumbar lordosis angle	2	0%	108	0.57 [−0.96, −0.19]	0%	Moderate^‡^
Average lateral deviation	2	50%	112	0.54 [−0.92, −0.16]	46%	Low^¶^
Quality of life	3	0%	138	0.73 [−1.07, −0.38]	0%	Moderate^‡^

GRADE, GRADE working group grades of evidence.

^†^Statistical heterogeneity results downgrade (*I* ^2^ > 25%). ^‡^Large confidence interval results downgrade. ^¶^Large confidence interval, statistical heterogeneity results downgrade.
